# Alcohol and other drug use among Aboriginal and Torres Strait Islander and non-Aboriginal and Torres Strait Islander men entering prison in New South Wales

**DOI:** 10.1186/s40352-015-0027-1

**Published:** 2015-08-11

**Authors:** Michael F Doyle, Tony G Butler, Anthony Shakeshaft, Jill Guthrie, Jo Reekie, Peter W Schofield

**Affiliations:** 1 Kirby Institute, UNSW Australia, High Street, Kensington, 2052 Australia; 2 National Drug and Alcohol Research Centre, UNSW Australia, High Street, Randwick, 2052 Australia; 3 National Centre for Indigenous Studies, Australian National University, 45 Sullivans Creek Road, Canberra, 2601 Australia; 4 School of Medicine and Public Health, The University of Newcastle, University Drive, Newcastle, 2308 Australia

**Keywords:** Alcohol, Illicit drug, Prisoners, Aboriginal, Treatment

## Abstract

**Introduction and aims:**

Prison entrants commonly have a history of problematic alcohol and other drug (AoD) use. Aboriginal and Torres Strait Islander (Indigenous) Australians are vastly overrepresented in Australian prisons with an incarceration rate 16 times that of non-Indigenous Australians. Relatively little attention has been given to the patterns of AoD use among prison entrants and we hypothesise that they may differ between Indigenous and non-Indigenous entrants. The aim of this paper is to compare the prior AoD use among Indigenous and non-Indigenous prison entrants and identify the implications for AoD treatment provision within prisons.

**Design and method:**

Cross-sectional random sample of 200 men recently received into New South Wales (NSW) criminal justice system.

**Results:**

During the 12 months prior to imprisonment, 106 prison entrants consumed alcohol at levels at which an intervention is recommended. Additionally during the four weeks prior to prison, 94 inmates had used illicit drugs daily. There was some overlap between these two groups; however, heroin users were less likely to consume alcohol at harmful levels. Relative to non-Indigenous entrants, Indigenous entrants prior to imprisonment used more cannabis but less amphetamine on a daily basis. There were no other significant differences between the alcohol or drug use of Indigenous and non-Indigenous prison entrants.

**Discussion and conclusion:**

Both Indigenous and non-Indigenous men entering prison have a history of high levels of AoD use but a slightly different treatment focus may be required for Indigenous inmates.

## Background

Problematic alcohol and other drug (AoD) use is common among those in prison with many inmates reporting they had been under the influence of alcohol and/or other drugs at the time of their offence (Butler et al. 2011a; Australian Institute of Health and Welfare 2013; Indig et al. 2010a). Nationally, just under half (46 %) of prison entrants reported consuming alcohol at ‘harmful’ levels (as defined by Alcohol Use Disorders Identification Test (Babor et al. 1992)) and 70 % had used illicit drugs once or more during the 12 months prior to prison (Australian Institute of Health and Welfare 2013). The 2009 New South Wales Inmate Health Survey reported that at the time of their current offence, 22 % of Indigenous men and one fifth of non-Indigenous men were intoxicated with alcohol, 29 % of Indigenous and 16 % of non-Indigenous men were under the influence of both alcohol and other (illicit) drugs, and 21 % of Indigenous and 22 % of non-Indigenous men were under the influence of illicit drugs only (Indig et al. 2010a). Over half (52 %) thought there was a link between their AoD use and their imprisonment (Indig et al. 2010a).

Injecting drug use, is widespread among prisons entrants with a large proportion (44 %) having previously injected and over half (56 %) having injected in the month prior to imprisonment (Butler et al. 2011a). In NSW methamphetamine was used daily/almost daily prior to prison by 14 % of men; crystalline methamphetamine (ice) was used daily/almost daily by 11.8 % of men; and heroin was used daily/almost daily by 8.5 % of male inmates (Indig et al. 2010a). Daily/almost daily illicit drug use (including injecting) in the year before prison was more common among Indigenous men (51 %) compared to non-Indigenous (38 %) with cannabis being the most commonly used illicit drug for both groups (Indig et al. 2010b). Despite the over-representation of illicit drug use among prisoners, relative to the general population, the most commonly used drug among inmates is nicotine; 83 % of Indigenous and 71 % non-Indigenous men reported being a current tobacco smoker (Indig et al. 2010a).

While high rates of AoD use in offender populations are well-established, the availability, uptake and efficacy of in-prison programs for these disorders is far less clear. In NSW half of the men with a history of AoD use had sought alcohol and or drug treatment prior to prison, with 61 % of this group stating they wanted help for their alcohol use problem (Indig et al. 2010a). Relative to this potential level of need alcohol treatment for people in prison is not common (Doyle et al. 2011), with a national survey of prisoner health reporting that only 17 % of Indigenous and 10 % of non-Indigenous inmates leaving prison had received treatment for problematic alcohol use (Australian Institute of Health and Welfare 2013).

Within Australian prisons there is a need to have a focus on Indigenous Australians as they are vastly overrepresented, making up 27 % of the prisoner population but only 2.5 % of the overall Australian population (Australian Bureau of Statistics 2014a; Australian Bureau of Statistics 2012). New South Wales has the largest Indigenous prisoner population with 2,492 (23.6 %) of the 10,566 Indigenous prisoners held in Australia’s prisons in 2014 (Australian Bureau of Statistics 2014a). The rate of Indigenous imprisonment further highlights the overrepresentation at 1,857 per 100,000 compared to 144 per 100,000 for non-Indigenous Australians (Australian Bureau of Statistics 2014a). Problematic use of AoD has been identified as a leading factor in the high rate of Indigenous imprisonment (Weatherburn 2008; Australian National Council on Drugs NIDaAC 2013), and first highlighted over 20 years ago by the 1991 Royal Commission into Aboriginal Deaths in Custody (Royal Commission into Aboriginal Deaths in Custody 1992). A number of strategies to address this nexus between Indigenous imprisonment and AoD use have been proposed (Australian National Council on Drugs NIDaAC 2013; Martire and Larney 2009). The strategies range from restrictions on alcohol supply in Indigenous communities to improved availability of treatment services and initiatives such as sobering up shelters as an alternative to police custody (Weatherburn 2008; Australian National Council on Drugs NIDaAC 2013; Royal Commission into Aboriginal Deaths in Custody 1992). Despite these initiatives the rate of imprisonment is higher in 2014 than it was at the time of the Royal Commission in 1991 (Doyle et al. 2011; Rodas et al. 2011).

One third of people entering prison are released within 12 months and over half are released after 24 months (Australian Bureau of Statistics 2014a). This window of opportunity suggests that effective screening for AoD problems on entry to prison is appropriate to enable treatment and referral pathways to be initiated during the incarceration period. There has been limited research in this area in Australia (Doyle et al. 2011), however, international evidence suggests that treatment for AoD disorders may be effective within prison (McGuire et al. 1991). It is unclear if the AoD treatment needs of prison entrants in Australia are being met, and the extent to which AoD problems and treatments ought to be tailored to the specific needs of Indigenous prisoners, such as levels of dependence severity. As far as we are aware, relatively little attention has been given to the patterns of AoD use among prison entrants. We hypothesise that those needs may differ between Indigenous and non-Indigenous entrants. Thus, this study aims to compare the prior AoD use among Indigenous and non-Indigenous prison entrants and identify the implications for AoD treatment provision in prisons.

## Methods

### Participants

The sample comprised 200 men recently received into the criminal justice system in the Hunter Region of New South Wales between September 2003 and June 2004. Full details of the study are published elsewhere, the data was collected as part of a study examining reported past Traumatic Brain Injury (TBI) (Schofield et al. 2006a; Schofield et al. 2006b; Perkes et al. 2011). Participants were randomly recruited and their TBI status was only determined after they had been recruited. The participants were recruited after being received into a police cell complex or a reception prison and included both those on remand for sentencing by the Courts and those recently sentenced. The project officer was primarily responsible for recruitment and due to resourcing, recruitment usually occurred one day per week. Either the project officer or a nurse within the prison reception unit administered the 11 page survey, with results being self-reported by participants. The project officer recruited 57 % of participants with only 3 % of those approached by them declining to take part, no refusal data was recorded by other recruiters. The cross checking of data showed similar participant responses between those interviewed by the project officer and other recruitment staff.

Recruitment was sequential; however, on days when resources did not permit sequential recruitment due to high volume of inmates entering custody, potential participants were identified by the last digit of their unique Corrective Services NSW assigned identification (ID) code in order from highest digit (nine) to lowest. In over 95 % of the cases the participant had been given this number when previously incarceration or arrest. Consequently, there was little likelihood of any association between the last digit of the number and the temporal sequence in which they had been received into custody for the current offence (Perkes et al. 2011; Butler and Allbutt 2003; Armand et al. 1997).

### Measures

Alcohol use was measured using the 10-item World Health Organization’s (WHO) Alcohol Use Disorders Identification Test (AUDIT) (Babor et al. 1992). Drug use questions asked about any, and daily, drug use in the past four weeks (nicotine, cannabis, heroin, amphetamines, prescribed medications). Any illicit drug use was defined as having used any or; anaesthetics, anabolic steroids, non-prescribed methadone or opioid other than heroin, heroin, cocaine, amphetamine (and other related stimulants), cannabis, hallucinogens, volatile solvents and volatile inhalants daily in the past four weeks.

Mental health status was assessed using the 10-item Kessler Psychological Distress Scale (K10), to measure levels of psychological distress (Butler et al. 2011b) and the International Personality Disorders Examination (IPDE), to measure impulsive personality (Fazel and Danesh 2002). IPDE is a screening tool used to detect mental health disorders and along with the K10 is widely used in epidemiological studies of mental health (Butler and Allbutt 2003). Other data reported are those recorded by the health staff when assessing inmate risk upon entry to prison, such as previous episodes of mental health treatment and self-harm episodes including previous suicide attempts. Details of previous TBI were reported as this was the main outcome measure of the original study (Schofield et al. 2006a; Schofield et al. 2006b). For this analysis TBI was defined as any injury ever to the head that caused a feeling of being ‘dazed or confused’ and or ‘loss of conciseness/blackout’. It was established that answers from these inmates were quite accurate as some results were cross checked using medical records (Kessler et al. 2003).

### Ethics

The study had ethics approval from Justice Health NSW and the Hunter New England Area Health Services’ Human Research Ethics Committees. Informed consent was required for participation.

### Statistical analysis

Participants were described by Indigenous status (yes/no), age (18–24 years, 25–39 years and 40+ years), marital status (married/defacto or single/separated), country of birth (Australia or other) and educational attainment (did not complete year 10, completed year 10, and completed year 12 or post school quantifications). For offending history, respondents were asked if they had been to juvenile detention or not. The primary offence for which they were in custody was categorised as being violent or non-violent, with a violent offence being one whereby harm was inflicted on another person.

Individual items on AUDIT are scored 0–4 and aggregated to a total from 0–40. Respondents’ scores were categorised two ways. First, using the standard WHO categories (Armand et al. 1997): 0 (no alcohol consumption); 1–7 (low-risk alcohol consumption); 8–19 (harmful/hazardous risk to health from alcohol consumption); and ≥20 (high-risk of harm from alcohol consumption and/or possibly alcohol dependent). Second, since WHO recommends an alcohol intervention for people who score ≥8, respondents’ AUDIT scores were categorised as either <8 (no treatment) and ≥8 (treatment recommended). Other drug use was categorised as yes, no or missing for daily use in the past 4 weeks.

For mental health status, each item on the IPDE was scored as positive or negative, with three or more positives in a single domain being an indication of that particular personality disorder (Armand et al. 1997). The K10 is scored numerically with a score of ≤19 indicating a minimal level of distress, 20 to 29 indicating an elevated level of distress, and ≥30 indicating a severe level of distress (Kessler et al. 2003). Only the most severe distress level category was used for analysis because entry to prison can of its own be a cause of distress. Answers to items for previous mental health treatment, suicide attempts, family member attempted suicide and self-harm episodes were categorised as yes, no or missing.

Data were analysed using IBMs’ software Statistical Package for the Social Sciences (SPSS) version 22. The characteristics of Indigenous and non-Indigenous participants were compared: Chi-square tests were used to compare categorical variables and Mann Whitney U tests for continuous variables. Logistic regression analysis was used to investigate factors associated with an AUDIT score of ≥8 (treatment recommended group). Variables with p < 0.1 level significance in univariate analysis were included in the multivariate model as well as Indigenous status and age.

## Results

### Inmate characteristics

Over half of the sample was aged between 25 and 39 years, 72 % were single, and 95 % were born in Australia (Table 1). One fifth (20 %) identified as being Indigenous, which reflects the Indigenous composition of the male prisoner population in NSW at the time of the study (Australian Bureau of Statistics 2014a). Educational attainment levels were similar between Indigenous and non-Indigenous inmates. For their current term of imprisonment, more Indigenous offenders (64 %) than non-Indigenous offenders (50 %) had committed offences categorised as violent. A similar number of Indigenous (80 %) and non-Indigenous (77 %) inmates scored 30 or over on the K10, indicating ‘severe’ distress. Two fifths (42 %) of non-Indigenous inmates and over half (53 %) of Indigenous inmates screened positive for impulsive personality (IPDE). There was a high prevalence of brain injury for both Indigenous and non-Indigenous participants, but there was not a statistically significant difference between the groups. Overall, none of the differences between Indigenous and non-Indigenous respondents were statistically significant.

**Table 1:** Demographic, offending history and mental health characteristics by Indigenous status

Characteristic	Indigenous (*n* = 40)	Non-Indigenous (*n* = 160)	Total	*P*-value
Age (years)	Median 28.7	Median 30.0		0.20^1^
	IQR 23 to 35	IQR 24 to 37		
Age category (years)				
18–24	14 (35.0 %)	43 (26.9 %)	57 (28.5 %)	0.36^2^
25–39	22 (55.0 %)	88 (55.0 %)	110 (55.0 %)	
40+	4 (10.0 %)	29 (18.1 %)	33 (16.5 %)	
Marital status				
Married/de facto	10 (25.0 %)	40 (25.0 %)	50 (25.0 %)	0.86^2^
Single/separated	27 (67.5 %)	116 (72.5 %)	143 (71.5 %)	
Missing	3 (7.5 %)	4 (2.5 %)	7 (3.5 %)	
Country of birth				
Australia	38 (95.0 %)	151 (94.4 %)	189 (94.5 %)	0.89^2^
Other	2 (5.0 %)	9 (5.6 %)	11 (5.5 %)	
Educational attainment				
Did not complete year 10	17 (42.5 %)	51 (31.9 %)	68 (34.0 %)	0.42^2^
Completed year 10	13 (32.5 %)	57 (35.6 %)	70 (35.0 %)	
HSC/Certificate/Degree	10 (25.0 %)	52 (32.5 %)	62 (31.0 %)	
Juvenile detention				
Yes	16 (40.0 %)	53 (33.1 %)	69 (34.5 %)	0.43^2^
No	24 (60.0 %)	106 (66.3 %)	130 (65.0 %)	
Missing	-	1 (0.6 %)	1 (0.5 %)	
Offence type				
Violent	25 (62.5 %)	80 (50.0 %)	105 (52.5 %)	0.10^2^
Non-violent	12 (30.0 %)	75 (46.9 %)	87 (43.5 %)	
Missing	3 (7.5 %)	5 (3.1 %)	8 (4.0 %)	
Number of arrests, Mean and median	Median 15.0	Median 10.0		0.06^1^
	IQR 1.0 to 7.5	IQR 1.0 to 11.0		
Kessler psychological distress scale (K10)				
No distress: 10–19	3 (7.5 %)	12 (7.5 %)	15 (7.5 %)	0.84^2^
Mild to moderate: 20–29	5 (12.5 %)	26 (16.2 %)	31 (15.5 %)	
Severe distress: 30+	32 (80.0 %)	122 (76.3 %)	154 (77.0 %)	
Impulsive personality (IPDE)				
Positive	21 (52.5 %)	67 (41.9 %)	88 (44.0 %)	0.23^2^
Negative	19 (47.5 %)	93 (58.1 %)	112 (56.0 %)	
Ever treated for a mental health problem				
Yes	13 (32.5 %)	48 (30.0 %)	61 (30.5 %)	0.76^2^
No	26 (65.0 %)	108 (67.5 %)	134 (67.0 %)	
Missing	1 (2.5 %)	4 (2.5 %)	5 (2.5 %)	
Have previously attempted suicide				
Yes	8 (20.0 %)	25 (15.6 %)	33 (16.5 %)	0.52^2^
No	31 (77.5 %)	130 (81.3 %)	161 (80.5 %)	
Missing	1 (2.5 %)	5 (3.1 %)	6 (3.0 %)	
Family member attempted suicide				
Yes	5 (12.5 %)	28 (17.6 %)	33 (16.5 %)	0.46^2^
No	33 (82.5 %)	126 (78.7 %)	159 (79.5 %)	
Missing	2 (5.0 %)	6 (3.7 %)	8 (4.0 %)	
Have previously self-harmed				
Yes	4 (10.0 %)	10 (6.2 %)	14 (7.0 %)	0.42^2^
No	35 (87.5 %)	144 (90.0 %)	179 (89.5 %)	
Missing	1 (2.5 %)	6 (3.8 %)	7 (3.5 %)	
Traumatic brain injury				
Yes	33 (82.5 %)	131 (81.9 %)	164 (82.0 %)	0.57^2^
No	7 (7.5 %)	29 (18.1 %)	36 (18.0 %)	

^1^Mann Whitney U Test

^2^Chi-square test

One quarter of non-Indigenous inmates reported that they did not consume alcohol in the 12 months prior to prison as indicated by an AUDIT score of 0 (Table 2). Over half of all Indigenous (55 %) and non-Indigenous (53 %) inmates scored ≥8 on the AUDIT, indicating a need for an alcohol intervention. Possible alcohol dependence was indicated among 22.5 % of both Indigenous and non-Indigenous respondents (scored ≥20 on AUDIT).

**Table 2:** Indigenous and non-Indigenous alcohol and daily illicit and licit drug use in the past 4 weeks

Alcohol and daily illicit and licit drug use		Indigenous (*N* = 40)	non-Indigenous (*N* = 160)	Chi-square p-value
No consumption	AUDIT 0	7 (17.5 %)	40 (25.0 %)	0.76
Low-risk	AUDIT 1–7	11 (27.5 %)	36 (22.5 %)	
Harmful/hazardous	AUDIT 8–19	13 (32.5 %)	48 (30.0 %)	
High-risk/dependent	AUDIT 20+	9 (22.5 %)	36 (22.5 %)	
Nicotine daily (drug) use past 4 weeks	Yes	33 (91.7 %)	117 (77.5 %)	0.06
	No	3 (8.3 %)	34 (22.5 %)	
	*Missing*	*4*	*9*	
Cannabis daily (drug) use past 4 weeks	Yes	17 (45.9 %)	55 (36.7 %)	0.05
	No	20 (54.1 %)	95 (63.3 %)	
	*Missing*	*3*	*10*	
Heroin daily (drug) use past 4 weeks	Yes	5 (13.2 %)	18 (12.3 %)	0.89
	No	33 (86.8 %)	128 (87.7 %)	
	*Missing*	*2*	*14*	
Amphetamine daily (drug) use past 4 weeks	Yes	1 (2.8 %)	24 (16.8 %)	0.03
	No	35 (97.2 %)	119 (83.2 %)	
	Missing	4	7	
Prescribed methadone/buprenorphine/naltrexone daily (drug) use past 4 weeks	Yes	3 (8.1 %)	11 (7.4 %)	0.88
	No	34 (91.9 %)	138 (92.7 %)	
	Missing	3	11	

Cannabis was the most common illicit drug used on a daily basis in the past 4 weeks, with statistically significantly greater use among Indigenous (46 %) than non-Indigenous (37 %) inmates (*p* = 0.05). Overall, compared with cannabis, considerably fewer inmates, both Indigenous and non-Indigenous, reported having used either amphetamine (14 %) or heroin (13 %). However, a statistically significantly smaller proportion of Indigenous inmates than non-Indigenous had used amphetamine on a daily basis in the past four weeks (3 % vs 17 %, *p* = 0.03). There were no statistically significant differences in the use of heroin or prescribed methadone/buprenorphine/naltrexone by Indigenous status.

### AoD use and mental health status

Although 24 % of respondents were alcohol abstinent, two thirds (64 %) of alcohol abstainers had consumed illicit drugs on a daily basis (Table 3). The majority of inmates had not been treated previously for a mental health problem, even though there were high levels of severe distress (K10) reported across all AUDIT categories. Three quarters of inmates reported daily nicotine use with high prevalence across all AUDIT categories.

**Table 3:** Alcohol, nicotine and illicit drug use, and mental health status by AUDIT category

Alcohol use	Daily nicotine use in past 4 weeks	Daily illicit^3^ drug in past 4 weeks	K10^4^ (‘severe’ distress)	Previously treated for mental health problem	Total
No consumption	32 (21.3 %)^1^	30 (31.9 %)	36 (23.4 %)	16 (26.2 %)	47 (23.5 %)
AUDIT = 0	(68.1 %)^2^	(63.8 %)	(76.6 %)	(34.0 %)	
Low-risk	35 (23.3 %)	22 (23.4 %)	41 (26.7 %)	13 (21.3 %)	47 (23.5 %)
AUDIT = 1 to 7	(74.5 %)	(46.8 %)	(87.2 %)	(27.7 %)	
Harmful/hazardous	50 (33.3 %)	23 (24.5 %)	47 (30.5 %)	15 (24.6 %)	61 (25 %)
AUDIT = 8 to 19	(82.0 %)	(37.7 %)	(77.0 %)	(24.6 %)	
High-risk/dependent	33 (22.0 %)	19 (20.2 %)	30 (19.5 %)	17 (27.9 %)	45 (22.5 %)
AUDIT = 20+	(73.3 %)	(42.2 %)	(66.7 %)	(37.8 %)	
Subtotal	150 (75 %)	94 (47.0 %)	154 (77 %)	61 (30.5 %)	200 (100 %)
Indicated no use	37	97	46^5^	144	-
Missing	13	9	-	5	-
Total	200	200	200	200	200

^1^Percentage within column

^2^Percentage within row

^3^Includes: Anaesthetics, anabolic steroids, non-prescribed methadone or opioids other than heroin, heroin, cocaine, amphetamine (and other related stimulants), cannabis, hallucinogens, volatile solvents and volatile inhalants

^4^Only severe distress was reported as entry to prison can be a distressing event

^5^Refers to a K10 score that did not indicate ‘severe distress level’

The univariate analysis showed that the odds of daily heroin use in the past four weeks were statistically significantly reduced (OR = 0.33, *p* = 0.02) among the alcohol treatment recommended group (Table 4). There was no significant difference between the treatment recommended and no treatment groups in their reported use of nicotine, cannabis and amphetamine in the past four weeks. The odds of the alcohol treatment recommended group using any illicit drug daily in the past four weeks were statistically significantly lower than the no treatment group (OR = 0.48, *p* = 0.01). However, when daily heroin use was excluded from the any illicit drug use category, the odds of reduced use by the alcohol treatment recommended group, compared to the no treatment group, were no longer significantly different (OR = 0.66, *p* = 0.15).

**Table 4:** Alcohol intervention and non-intervention groups by demographics/health issue

Demographic/Health issue^1^		AUDIT < 8	AUDIT ≥8		*P*-value	Multivariate O.R. (95 % CI)	*P*-value
		(no treatment)	(treatment recommended)	Univariate			
		(n = 94)	(n = 106)	O.R. (95 % CI)			
Indigenous status	Indigenous	18 (19.1 %)	22(20.8 %)	1.0		1.0	
	Non-Indigenous	76 (80.9 %)	84 (79.2 %)	0.90 (0.45–1.81)	0.78	0.79 (0.37–1.66)	0.53
Age in years	18–24	25 (25.6 %)	32 (30.2 %)	1.0	0.78	1.0	
	25–39	52 (55.3 %)	58 (54.7 %)	0.87 (0.46–1.66)	0.68	0.93 (0.47–1.87)	0.84
	40+	17 (18.1 %)	16 (15.1 %)	0.75 (0.31–1.74)	0.48	0.67 (0.27–1.67)	0.39
Traumatic brain injury	Yes	74 (78.7 %)	90 (84.9 %)	1.0			
	No	20 (21.3 %)	16 (15.1 %)	0.66 (0.32–1.22)	0.26		
	missing	0	0				
Daily nicotine use in past 4 weeks	Yes	67 (77.9 %)	83 (82.2 %)	1.0			
	No	19 (22.1 %)	18 (17.8 %)	0.76 (0.37–1.57)	0.47		
	*Missing*	8	5				
Daily cannabis use in past 4 weeks	Yes	38 (44.7 %)	37 (36.3 %)	1.0			
	No	47 (55.3 %)	65 (63.7 %)	0.70 (0.39–1.27)	0.24		
	*Missing*	9	4				
Daily heroin use in past 4 weeks	Yes	16 (18.8 %)	7 (7.1 %)	1.0		1.0	
	No	69 (81.2 %)	92 (92.3 %)	0.33 (0.13–0.84)	0.02	0.37 (0.14–0.96)	0.04
	*Missing*	9	7				
Daily amphetamine use in past 4 weeks	Yes	14 (17.3 %)	11 (11.2 %)	1.0			
	No	67 (82.7 %)	87 (88.8 %)	0.60 (0.26–0.26)	0.25		
	*Missing*	13	8				
Daily prescribed methadone/buprenorphine/naltrexone in past 4 weeks	Yes	5 (5.7 %)	5 (4.9 %)	1.0			
	No	82 (94.3 %)	98 (95.1 %)	0.44 (0.14–1.37)	0.16		
	*Missing*	7	3				
Any illicit drug use daily (excl. heroin)	Yes	45 (51.1 %)	42 (40.8 %)	1.0		1.0	
	No	43 (48.9 %)	61 (59.2 %)	0.66 (0.37–1.17)	0.15	0.64 (0.35–1.18)	0.15
	*Missing*	6	3				
Any illicit drug use daily	Yes	52 (59.1 %)	42 (40.8 %)	1.0			
	No	36 (40.1 %)	61 (59.2 %)	0.48 (0.27–0.80)	0.01		
	*Missing*	6	3				

^1^No statistically significant differences between the AUDIT identified no treatment and treatment recommended groups by demographic characteristics of marital status, country of birth, educational attainment, juvenile detention , offence type, K10, Impulsive personality, even been treated for a mental health problem, have previously attempted suicide, family member attempted suicide, and have previously self-harmed. Data not shown

The multivariate analysis showed that the odds of heroin use by those in the alcohol treatment recommended group remained significantly lower (OR = 0.37, *p* = 0.04) among those who had used heroin daily when Indigenous status, age and any illicit drug use (excluding heroin) are factored into the model. There was no statistically significant association between the treatment recommended group and Indigenous status, age, TBI or drug use (excluding heroin).

## Discussion

Based on the AUDIT scores, over half (106) of the sample met the criteria for requiring an alcohol intervention and 45/106 (43 %) of that group warranted further investigation for possible alcohol dependence. We found no significant differences between Indigenous and non-Indigenous inmates in regard to alcohol use, suggesting that problematic alcohol use is equally spread between these two groups. These results would imply that about 50 % of prison entrants could benefit from an alcohol intervention and that supervised withdrawal from alcohol may be required for between 20 and 25 % of prison entrants. The extent to which case management occurs for alcohol use disorders in Australian prisons is unknown.

Illicit drug use was common among inmates, with almost half reporting daily use. Inmates who reported using heroin on a daily basis either consumed less alcohol or no alcohol. The major differences by Indigenous status were that Indigenous inmates were more likely to use cannabis (*p* = 0.05), but less likely to use amphetamine on a daily basis than non-Indigenous inmates (*p* = 0.03). Tobacco use was high among Indigenous and non-Indigenous inmates with 150/200 (75 %) smoking on a daily basis implying a role for smoking cessation interventions. Of the 200 study participants, based on our screening measures, only 42 (21 %) did not merit any AoD behavioural treatment, 64/200 (32 %) warranted an alcohol (but not illicit drug) intervention; 52/200 (26 %) required help for illicit drug use (but not alcohol) and 42/200 (21 %) required assistance for both alcohol and illicit drug use. Despite these differences it is likely that if these inmates were to receive an AoD intervention program, that program would be focused on illicit drug use only or alcohol and illicit drug use, but not focused on alcohol specifically, as discussed further below.

The IPDE scores indicated that 44 % of inmates potentially had impulsive personalities and the K10 results showed that 77 % (*n* =154) had severe psychological distress. The K10 result, , should be interpreted with caution as entry to prison can be a distressing event but, nonetheless, the findings here are broadly consistent with the well-established high levels of poor mental health among people in prison (Butler et al. 2011b; Fazel and Danesh 2002). Both the IPDE and the K10 are screening tests and further assessment is required before a diagnosis can be made, but it is highly likely that a significant proportion of the participants in this study would benefit from support for their mental health.

Alcohol and other drug treatment needs for Indigenous and non-Indigenous prison entrants may be different. The results indicate that there is some scope for recommending a focus on cannabis among Indigenous inmates, as more Indigenous than non-Indigenous men reported daily use (46 % versus 37 %, *p* = 0.05). Other data supports a focus on cannabis use for Indigenous Australians not just in prison but in the general population. The National Drug Household Survey (2011) reported cannabis use among Indigenous respondents was 19 % versus 10 % for non-Indigenous respondents (age of ≥14) (Australian Institute of Health and Wealfare 2011), and the National Aboriginal and Torres Strait Islander Health Survey reported that one in five (19 %) of Indigenous respondents (aged ≥15) had used cannabis in the previous 12 months (no comparative figure for non-Indigenous use) (Australian Bureau of Statistics 2014b). However, within Australian prisons there appears to be no cannabis specific programs for either Indigenous or non-Indigenous inmates (Doyle et al. 2011; Rodas et al. 2011), even though it is the most commonly used illicit drug among prison inmates (Indig et al. 2010a).

Alcohol and other drug treatment needs of prison entrants are possibly different from those of other inmates. Within this study there was some difference in AoD use relative to the findings of the 2009 NSW Inmate Health Survey (Indig et al. 2010a). For example, based on AUDIT scores, an intervention for alcohol use would have been indicated for 53 % in this sample but for 63 % of respondents in the Inmate Health Survey. There could be a number of factors as to why such a difference occurred, including 3 years difference between the data collection dates. Another notable difference is that the participants for this sample are all prison entrants from one site, while the Inmate Health Survey represents a cross section of the whole prisoner population in NSW, with many of those inmates having been in prison for 12 months or more. Another possibility is a difference in recall, which is a strength of this study as participants in this survey were asked to recall recent use of AoD rather than recalling AoD use that occurred several months or even years earlier. With this group participants some caution is due when interpreting results as prison entrants could possibly be reluctant to answer questions that relate to criminal activity (i.e. consuming illicit drugs). However, inmates responses in this study had a high degree of consistency with the notes recorded in their medical records and as such can be thought to be fairly accurate with their responses (Schofield et al. 2011). Compared to the 2009 NSW Inmate health Survey, the numbers in this study, particularly of Indigenous participants limits the statistical power.

Court based and mandated referral pathways into drug treatment occur regularly. Drug Courts operate in every Australia jurisdiction, but the national response to the most commonly used substance, alcohol has been much less coordinated (Payne et al. 2008). Alcohol has been included as an extension of the Drug Court in some, but not all, Australian jurisdictions (Payne et al. 2008). This extension is used predominantly in areas that have a higher proportion of Indigenous Australians as residents, which is predominantly away from the large state capital cities and major population centres (Payne et al. 2008). These results demonstrate that more than half of this sample may benefit from an alcohol intervention and that non-Indigenous men are in equal need of an alcohol related intervention.

There is limited aggregated data on the provision of AoD treatment within prison but yet there are prison based AoD programs operating in every Australian jurisdiction (Doyle et al. 2011; Rodas et al. 2011). It is not known how many inmates commence and complete these, nor is it known how long the average wait time from prison entry and assessment to commencement of an AoD treatment program. There appears to be no published research into the long term outcomes of those who complete the AoD programs and it is not known if people who undertake these programs are any less likely to return to prison. With such sparse research into prison based AoD treatment it is not known if Indigenous inmates have different outcomes to others, what is clear is that there are few jurisdictions that have Indigenous specific programs. Further research in this area is essential, particularly with the view to improving services for Indigenous Australians who are vastly over represented in the Nations prisons.

Alcohol and other drug treatment in prison can be effective, but such treatment should be specific to the individual’s needs or it can be harmful (McGuire et al. 1991; Office of the Insepector General of Custodial Services 2008). Prison-based AoD treatment programs are operated in all states and territories in Australia. Inmates are assessed by staff and are referred to or placed into the AoD treatment programs (Doyle et al. 2011; Rodas et al. 2011). There are different AoD programs which have different focuses, but generally program classes consist of about up to 12 to 20 inmates attending a one to two hour class two to three times a week for around 12 weeks (Doyle et al. 2011; Rodas et al. 2011). Limited aggregated data are available on the number of inmates that undertake and complete AoD treatment programs and it is not known how long a wait there is between entry to prison and commencement of an AoD program (Doyle et al. 2011; Rodas et al. 2011). What is quite clear, however, is that few of these programs are specifically alcohol focused, most are for alcohol and other drug use, and few AoD programs are specifically for Indigenous people (Doyle et al. 2011; Rodas et al. 2011). This research indicates that while both Indigenous and non-Indigenous prison entrants would benefit from AoD treatment there is possibly a need for different focuses between these groups.

## References

[CR1] Armand W, Loranger AW, Janca A, Sartorius N (1997). Assessment and Diagnosis of Personality Disorders : The ICD-10 International Personality Disorder Examination (IPDE).

[CR2] Australian Bureau of Statistics (2012). 2011 Census of Population and Housing - Counts of Aboriginal and Torres Strait Islander Australians : 4713.0.

[CR3] Australian Bureau of Statistics (2014). Prisoners in Australia 2014 : 4517.0.

[CR4] Australian Bureau of Statistics (2014). Australian Aboriginal and Torres Strait Islander Health Survey: updated results 2012–13.

[CR5] Australian Institute of Health and Wealfare (2011). National Drug Household Survey 2010.

[CR6] Australian Institute of Health and Welfare (2013). The Health of Australia’s Prisoners 2012.

[CR7] Australian National Council on Drugs NIDaAC (2013). Bridges and Barriers: Addressing Indigenous Incarceration and Health: Revised Edition.

[CR8] Babor TF, Higgins-Biddle JC, Saunders JB, Monteiro MG (1992). Alcohol Use Disorders Identification Test (AUDIT).

[CR9] Butler TG, Allbutt S (2003). Mental Illness Among New South Wales’ Prisoners Sydney.

[CR10] Butler TG, Lim D, Callander D (2011). National Prison Entrants’ Bloodborne Virus and Risk Behaviour Survey Report 2004, 2007, and 2010.

[CR11] Butler TG, Indig D, Allnutt S, Mamoon H (2011). Co-occurring mental illness and substance use disorder among Australian prisoners. Drug and Alcohol Review.

[CR12] Doyle MF, Fisher C, Saggers S (2011). Alcohol Intervention Programs within Australian Prisons for Aboriginal and Torres Strait Islander Men.

[CR13] Fazel S, Danesh J (2002). Serious mental disorder in 23,000 prisoners: a systematic review of 62 surveys. Lancet.

[CR14] Indig D, Topp L, Ross B, Mamoon H, Border B, Kumar S (2010). 2009 NSW Inmate Health Survey: Key Findings Report.

[CR15] Indig D, McEntyre E, Page J (2010). 2009 NSW Inmate Health Survey: Aboriginal Health Report.

[CR16] Kessler RC, Baker PR, Colpe LJ, Epstein JF, Gfroerer JC, Hiripi E (2003). Screening for serious mental illness in the general population. Archives of General Psychiatry.

[CR17] Martire KA, Larney S (2009). Aboriginal participation in Magistrate Early Referral into Treatment Program (Drug Court NSW).

[CR18] McGuire J, Priestley P, Andrews D, Lipsey MW, Losel F, Knott C (1991). What Works: Reducing Re-offending (Guidelines from Research and Practice).

[CR19] Office of the Insepector General of Custodial Services (2008). Report into the review of assessment and classification within the Department of Corrective Services.

[CR20] Payne J, Kwiatkowski M, Wundersitz J (2008). Police Drug Diversion: A Study of Criminal Offending Outcomes.

[CR21] Perkes I, Schofield PW, Butler TG, Hollis SJ (2011). Traumatic brain injury rates and sequelae: a comparison of prisoners with a matched community sample in Australia. Brain Injury.

[CR22] Rodas A, Bode A, Dolan K (2011). Supply, Demand and Harm Reduction Strategies in Australian Prisons: an update.

[CR23] Royal Commission into Aboriginal Deaths in Custody. (1992). *Diversion from Police Custody; Particularly from Arrests For Drunkenness {Recommendations 79–88}.* Canberra, Australia: Australian Government.

[CR24] Schofield PW, Butler TG, Hollis SJ, Smith NE, Lee SJ, Kelso WM (2006). Neuropsychiatric Correlates of Traumatic Brain Injury (TBI) among Australian Prison Entrants. Brain Injury.

[CR25] Schofield PW, Butler TG, Hollis SJ, Smith NE, Lee SJ, Kelso WM (2006). Traumatic brain injury among Australian prisoners: rates, recurrence and sequelae. Brain Injury.

[CR26] Schofield PW, Butler TG, Hollis SJ, D’Este C (2011). Are prisoners reliable survey respondents? A validation of self-reported traumatic brain injury (TBI) against hospital medical records. Brain Injury.

[CR27] Weatherburn D (2008). Role of drug and alcohol policy in reducing indigenous over-representation in prison. The Drug and Alcohol Review.

